# Seroprevalence and regional risk assessment of bluetongue virus among livestock in Central Saudi Arabia

**DOI:** 10.14202/vetworld.2025.2106-2112

**Published:** 2025-07-30

**Authors:** Roua A. Alsubki

**Affiliations:** Department of Clinical Laboratory Science, Chair of Medical and Molecular Genetics Research, College of Applied Medical Science, King Saud University, Riyadh, Saudi Arabia

**Keywords:** bluetongue virus, camels, cattle, *Culicoides*, enzyme-linked immunosorbent assay, goats, livestock, Saudi Arabia, seroprevalence

## Abstract

**Background and Aim:**

Bluetongue virus (BTV), an arbovirus of major economic importance, affects domestic and wild ruminants globally and is primarily transmitted by Culicoides biting midges. The virus is endemic in many regions, yet limited data are available for Saudi Arabia. This study aimed to determine the seroprevalence of BTV antibodies in cattle, goats, sheep, and camels across two ecologically distinct regions in central Saudi Arabia and to assess species- and region-specific risk profiles.

**Materials and Methods:**

A total of 1,194 serum samples were collected from apparently healthy livestock (280 cattle, 159 camels, 429 sheep, and 326 goats) in Riyadh and Al-Qassim between October 2023 and March 2024. Samples were tested for BTV antibodies using a commercial competitive enzyme-linked immunosorbent assay. Statistical analysis included Chi-square tests and odds ratios with 95% confidence intervals to compare prevalence rates between species and regions.

**Results:**

The overall BTV seroprevalence was 44.6% (533/1,194). Goats had the highest prevalence (59.8%), followed by cattle (51%), sheep (36.3%), and camels (22.6%). In Riyadh, cattle (55.7%) and goats (55%) showed the highest rates, while in Al-Qassim, goats (65.7%) were most affected. Camels consistently showed the lowest seroprevalence (18.6%–25%). Statistically significant differences in seroprevalence were observed among species and between regions (p < 0.05).

**Conclusion:**

BTV is endemic in central Saudi Arabia, with substantial species and regional variability. Goats and cattle are at higher risk, indicating a need for species-targeted surveillance and vector control. The findings support the implementation of national bluetongue monitoring strategies and lay the groundwork for future molecular and longitudinal studies.

## INTRODUCTION

Bluetongue virus (BTV) is a globally significant arbovirus belonging to the genus *Orbivirus* within the family *Reoviridae*. To date, 29 distinct serotypes of BTV have been identified, with ongoing research continuing to report newly emerging variants [1, 2]. The virus primarily infects domestic and wild ruminants, especially sheep, goats, cattle, and camels. Among these, sheep are the most clinically susceptible species [3–5]. BTV is transmitted chiefly through the bites of infected *Culicoides* midges, which act as biological vectors [6]. Clinical manifestations of bluetongue (BT) disease include fever, oral ulcerations, nasal discharge, facial and lip edema, and, in severe cases, mortality [7]. While sheep typically develop severe disease, cattle and other ruminants often serve as reservoir hosts, exhibiting mild or subclinical symptoms [8]. The economic implications of BTV are considerable due to reduced productivity, trade restrictions, and high costs associated with disease management [9, 10].

The global distribution of BTV closely mirrors the habitat of competent *Culicoides* vectors, whose population dynamics are influenced by climatic variables such as temperature, humidity, and precipitation [11, 12]. In recent decades, the geographical spread of BTV has significantly widened, driven by climate change, globalization, and the movement of infected animals or vectors through international trade and transportation networks [9, 13]. This expansion has led to the emergence of BTV in previously non-endemic regions, including parts of Europe, Asia, and the Middle East, posing substantial health and economic risks to naïve ruminant populations [14, 15].

BTV is considered endemic across much of the African continent, although comprehensive prevalence data are still lacking for many regions [16]. Post-rainfall outbreaks have been reported in cattle, sheep, and goats in North and East African countries, such as Egypt, Algeria, Tunisia, and Kenya [17, 18]. Southern Africa has also reported widespread outbreaks in small ruminants, including in Botswana, Lesotho, Madagascar, Namibia, South Africa, and Zimbabwe [4, 19–22]. However, detailed serotype information remains largely confined to South Africa – which has reported serotypes 1 through 24 – and Malawi, where multiple serotypes such as 1, 2, 3, 5, 8, 10, 15, 20, 21, and 22 have been documented [21]. In Europe, BTV emergence has been observed since the late 20^th^ century, likely originating from North Africa and spreading into Southern Europe [22–24].

In Saudi Arabia, the livestock industry plays a pivotal role in the agricultural economy, encompassing large populations of sheep, goats, cattle, and camels [25]. The country’s climatic conditions, characterized by high temperatures and seasonal rainfall, are conducive to the transmission of BTV by *Culicoides* vectors [26–28]. Seroprevalence studies from nearby Middle Eastern countries – including the United Arab Emirates, Oman, and Iraq – have reported varying levels of BTV antibodies, reflecting ongoing or previous circulation of the virus within the region [29–32].

In 2012, a national study on BTV seroprevalence in Saudi Arabia found significant levels of exposure across various livestock species. Reported seroprevalence rates included 54.1% in sheep, 53.3% in goats, 44.8% in cattle, and 25.7% in camels. These results highlight the widespread circulation of the virus. Regions with elevated seroprevalence were typically characterized by environmental conditions favorable to the breeding of *Culicoides* vectors, such as high temperatures and intermittent rainfall [28]. Collectively, these findings affirm the endemic presence of BTV in Saudi Arabia and underscore the importance of sustained surveillance, integrated vector management, and species-specific disease control strategies to mitigate the virus’s health and economic impacts.

The present study aimed to determine the seroprevalence of BTV antibodies among major livestock species – cattle, goats, sheep, and camels – in two ecologically distinct regions of central Saudi Arabia: Riyadh and Al-Qassim. By utilizing a validated competitive enzyme-linked immunosorbent assay (cELISA) Kit (IDEXX Bluetongue Competition Ab, IDEXX, USA; Catalog No. P00450), targeting the BTV viral protein 7 (VP7) antigen, the study sought to provide robust estimates of exposure levels across species and regions. Furthermore, the research aimed to identify species-specific and regional variations in seroprevalence, evaluate potential risk patterns, and assess the need for species-targeted surveillance and control interventions. The findings are intended to support national efforts in disease monitoring, risk mitigation, and the development of regionally adapted BT control strategies within the Kingdom.

## MATERIALS AND METHODS

### Ethical approval

All procedures involving animals were conducted in compliance with the animal welfare code of Saudi Arabia. Ethical approval for this study was granted by the Scientific Committee of the Animal Sector, Ministry of Environment, Water, and Agriculture, in April 2023 (Approval No. 09-1445).

### Study period and location

The study was carried out between October 2023 and March 2024 in two regions of Saudi Arabia: Riyadh (central) and Al-Qassim (north-central). These areas were selected due to their substantial livestock populations and distinct climatic conditions, which are known to influence *Culicoides* vector activity and, consequently, BTV transmission dynamics.

### Study population and sampling design

The sample sizes were designed using Stephen Thompson’s formula to ensure a 95% confidence level for detecting a minimum prevalence of 10%.

The study focused on four livestock species commonly raised in Saudi Arabia: cattle, camels, sheep, and goats – all of which are known to be susceptible to BTV infection. A total of 1,194 blood samples were collected from clinically healthy animals across both regions using simple random sampling from farms, livestock markets, and slaughterhouses.

- Cattle**:** 280 samples (158 from Riyadh; 122 from Al-Qassim)
- Camels**:** 159 samples (100 from Riyadh; 59 from Al-Qassim)
- Sheep**:** 429 samples (226 from Riyadh; 203 from Al-Qassim)
- Goats**:** 326 samples (180 from Riyadh; 146 from Al-Qassim).

### Blood collection and serum preparation

Approximately 10 mL of blood was aseptically drawn from the jugular vein of each animal using sterile vacutainers. Samples were centrifuged at 1,000 × *g* for 10 min to separate the serum, which was then aliquoted and stored at −20°C until testing.

### Serological testing

Serum samples were analyzed using a cELISA kit (IDEXX Bluetongue Competition Ab, IDEXX, USA; Catalog No. P00450), following the manufacturer’s protocol. The assay has a reported specificity of 100% and a sensitivity of 82.8%.

The procedure was as follows:

- Serum samples and controls were added to microplate wells pre-coated with recombinant VP7 antigen and incubated for 45 min at room temperature (21°C ± 5°C).
- Without washing, 100 μL of horseradish peroxidase-conjugated anti-VP7 monoclonal antibody was added, followed by a second 45-min incubation.
- After three washes, 100 μL of tetramethylbenzidine substrate was added and incubated in the dark for 10 min.
- The reaction was stopped by adding 100 μL of stop solution.
- Optical density (OD) was read at 450 nm using an AMR-100 microplate reader (Allsheng, China).

#### Interpretation of results

OD ≤70% of the mean negative control: Positive

OD ≥80% of the mean negative control: Negative

OD between >70% and <80%: Doubtful (retested as per protocol) [33, 34].

### Statistical analysis

Descriptive statistics were used to estimate the seroprevalence of BTV antibodies across species and regions. Differences in prevalence between species and regions were evaluated using Chi-square (χ^2^) tests (Statistical Package for the Social Sciences v.26, IBM Corp., NY, USA). Odds ratios with 95% confidence intervals were calculated using R statistical software version 4.2.2 (R Core Team, Vienna, Austria) to determine the strength of association between regional location and BTV seropositivity within each species. A p < 0.05 was considered statistically significant.

## RESULTS

### Overall seroprevalence by species

A total of 1,194 serum samples were analyzed to determine the seroprevalence of BTV across four livestock species: 280 from cattle, 159 from camels, 429 from sheep, and 326 from goats. The overall BTV seroprevalence among all tested animals was 44.6% (533/1,194).

**Figure 1:**
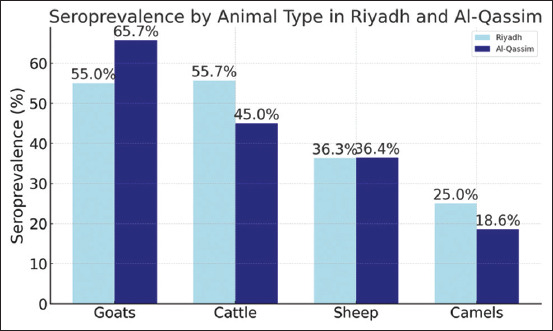
Comparative seroprevalence of bluetongue virus by livestock species and region (Riyadh and Al-Qassim) in Saudi Arabia.

Goats exhibited the highest overall seropreva-lence at 59.8% (195/326), followed by cattle at 51% (143/280). Sheep demonstrated a moderate seroprevalence of 36.3% (156/429), while camels had the lowest rate at 22.6% (36/159) (Table 1 and Figure 1). These results indicate that goats and cattle are at a higher risk of BTV exposure compared to sheep and camels.

**Table 1:** BTV seroprevalence by species and region with statistical analysis.

Species	Region	Number of samples	Positive (%)	Chi-square (vs. Other species)	p-value	Odds ratio (95% CI)	Regional comparison (χ²/p-value)
Cattle	Riyadh	158	88 (55.7)	16.32 (vs. Sheep)	<0.001*	1.54 (1.01–2.36) (Riyadh vs. Al-Qassim)	χ^2^ = 3.89, p = 0.049*
	Al-Qassim	122	55 (45)	5.21 (vs. Goats)	0.022*		
Goats	Riyadh	180	99 (55)	72.45 (vs. Camels)	<0.001*	1.57 (1.05–2.35) (Al-Qassim vs. Riyadh)	χ^2^ = 4.76, p = 0.029*
	Al-Qassim	146	96 (65.7)				
Sheep	Riyadh	226	82 (36.3)	0.01 (vs. Al-Qassim sheep)	0.920	—	χ^2^ = 0.01, p = 0.920
	Al-Qassim	203	74 (36.4)				
Camels	Riyadh	100	25 (25)	1.12 (vs. Al-Qassim camels)	0.290	—	χ^2^ = 1.12, p = 0.290
	Al-Qassim	59	11 (18.6)				
Total	—	1,194	533 (44.6)	—	—	—	—

χ^2^ and p-values for species-wise comparisons (e.g., cattle vs. sheep). Odds ratios (OR) for regional differences (e.g., goats in Al-Qassim vs. Riyadh). BTV=Bluetongue virus

### Species-specific seroprevalence in Riyadh

In Riyadh, cattle showed the highest BTV seroprevalence at 55.7% (88/158), closely followed by goats at 55% (99/180). Sheep had a seroprevalence of 36.3% (82/226), and camels recorded the lowest at 25% (25/100). These findings suggest that cattle and goats in Riyadh are more likely to have been exposed to BTV compared to other species in the same region.

### Species-specific seroprevalence in Al-Qassim

In Al-Qassim, goats again demonstrated the highest seroprevalence, with 65.7% (96/146) testing positive. Cattle showed a seroprevalence of 45% (55/122), while sheep had a seroprevalence of 36.4% (74/203). Camels again had the lowest rate at 18.6% (11/59). These results are consistent with the pattern observed in Riyadh, emphasizing the higher susceptibility of goats and cattle to BTV infection.

### Regional comparison of species seroprevalence

A comparison between regions revealed notable differences in species-specific seroprevalence. Goats in Al-Qassim had a significantly higher seroprevalence (65.7%, 96/146) compared to those in Riyadh (55%, 99/180). Conversely, cattle in Riyadh exhibited a higher seroprevalence (55.7%, 88/158) than those in Al-Qassim (45%, 55/122). In contrast, sheep and camels displayed relatively stable seroprevalence rates across regions, with camels consistently demonstrating the lowest levels of BTV antibodies [(18.6%, 11/59) and 25% (25/100)].

### Summary of observations

The findings indicate that cattle and goats are the most affected livestock species, with seroprevalence rates consistently exceeding 50% in both regions. Conversely, camels exhibited the lowest levels of exposure, potentially due to lower susceptibility, distinct immune responses, or reduced exposure to infected *Culicoides* vectors. The observed regional differences in BTV seroprevalence may be attributed to environmental variations, differences in vector abundance, and variations in livestock management practices.

## DISCUSSION

### Global and regional importance of BTV

BTV continues to impose a substantial economic burden on the global livestock industry through reduced productivity, trade limitations, and increased mortality, particularly in clinically susceptible species such as sheep [7, 10]. The virus remains a key emerging arboviral threat, affecting both animal health and international trade. Over the past few decades, the epidemiology of BTV has shifted significantly, driven largely by climate change and ecological disruption, which influence the abundance and distribution of *Culicoides* vectors [11, 12]. These changes have facilitated the emergence of BTV in regions previously considered non-endemic and have contributed to the introduction and spread of novel serotypes across different geographical zones [5, 6, 9].

### Shifting epidemiology and challenges in the Middle East

The dynamic distribution of BTV has posed new challenges for ruminant health management, early diagnosis, and regulatory preparedness [15, 35]. In the Middle East – including Saudi Arabia – there is a notable scarcity of contemporary epidemiological data on BTV, hindering risk assessment and effective control planning. Moreover, the absence of coordinated animal disease monitoring systems and vector surveillance networks in the region elevates the risk of uncontrolled transmission. To bridge these gaps, this study provides updated sero-epidemiological insights into BTV circulation in two central regions of Saudi Arabia.

### High seroprevalence indicates endemic circulation

This study revealed a high overall BTV seroprevalence of 44.6% in livestock, with marked variations among species and regions. These findings confirm BTV’s endemic status in Saudi Arabia, consistent with earlier reports from the Middle East [26, 28]. The results provide important species- and region-specific data, contributing to a broader understanding of arboviral transmission dynamics in arid and semi-arid environments. The data also reinforce the need to include BTV in ongoing surveillance and control programs in the Kingdom.

### Goats and cattle as high-risk hosts

Among the species tested, goats exhibited the highest seroprevalence (59.8%), followed by cattle (51%), sheep (36.3%), and camels (22.6%). These trends are consistent with observations from neighboring countries, such as Oman, where small ruminants – especially goats – showed high BTV antibody prevalence [29]. The elevated seroprevalence in cattle may be attributed to their longer lifespan and greater cumulative exposure to *Culicoides* vectors. Goats’ heightened susceptibility may also stem from environmental factors such as irrigation systems and intensive farming in areas like Al-Qassim, which support vector breeding habitats [11].

### Regional differences in exposure

Regional comparisons further emphasized variation in exposure risk. Goats in Al-Qassim had the highest seroprevalence (65.7%, 96/146), possibly reflecting favorable environmental conditions for vector proliferation, such as increased humidity and agricultural irrigation. In contrast, cattle in Riyadh exhibited a higher seroprevalence (55.7%, 88/158) than their counterparts in Al-Qassim (45%, 55/122), potentially due to differences in livestock movement, density, or management practices. Camels consistently showed the lowest seroprevalence [(18.6%, 11/59) and (25%, 25/100)] across both regions, suggesting lower susceptibility or reduced vector-host interaction.

### Factors limiting BTV seroprevalence in camels and sheep

The relatively low seroprevalence in camels aligns with earlier findings from Saudi Arabia and African countries [26, 28, 36]. This could be due to several factors, including species-specific immunity, vector feeding preferences, or the lower likelihood of camels co-mingling with more susceptible ruminants. Although sheep are known to be the most clinically affected by BTV, their lower antibody prevalence (36.3%, 82/226) may reflect shorter lifespans, less frequent exposure to infected vectors, or more protective husbandry practices that reduce their contact with vectors.

### Comparative evidence from other countries

Studies conducted in other geographic settings further support the multifactorial risk of BTV exposure. In Italy, BTV seroprevalence in cattle and buffalo reached 43.6% and 85.4%, respectively, with older age and high temperatures as risk factors [37]. A national survey in Peru identified warmer temperatures and lower altitudes as contributors to elevated seroprevalence in domestic ruminants [38]. In Egypt, exposure in sheep and goats was significantly associated with female sex, older age, and a history of abortion [39]. These findings underscore the importance of region-specific risk assessments in developing BTV prevention strategies.

### Diagnostic considerations and limitations

The 44.6% (553/1,194) seroprevalence reported in this study closely mirrors historical data from Saudi Arabia, such as the 47.3% reported in 2012 [28], potentially reflecting persistent transmission supported by evolving environmental conditions and BTV strains. However, the sensitivity of the cELISA used (82.8%) may result in a slight underestimation of true prevalence due to potential false negatives [40, 41]. These limitations should be considered when interpreting the data.

### Need for vector surveillance and future research

To date, no formal *Culicoides* surveillance system exists in Saudi Arabia, which presents a significant gap in understanding the seasonality and distribution of BTV vectors. Integrating entomological monitoring with serological surveys would offer a more comprehensive picture of disease dynamics. Furthermore, the study’s reliance on antibody detection limits its ability to assess active infections or identify circulating serotypes. Future research should incorporate molecular techniques for viral genotyping and longitudinal studies to assess the impact of climate change and animal movement on the emergence of vector-borne diseases.

## CONCLUSION

This study provides compelling evidence for the widespread circulation of BTV among livestock in central regions of Saudi Arabia. The overall seroprevalence was 44.6% (553/1,194), with goats (59.8%, 193/326) and cattle (51%, 143/280) exhibiting the highest exposure rates, followed by sheep (36.3%, 156/429) and camels (22.6%, 36/159). Regional analysis revealed significant variability, with goats in Al-Qassim and cattle in Riyadh being particularly affected. These findings highlight species- and region-specific susceptibility patterns that are crucial for informing targeted disease control strategies.

From a practical standpoint, the results emphasize the need for integrated BTV surveillance programs, especially in ecologically vulnerable regions such as Al-Qassim and Riyadh. Priority should be given to goats and cattle, which appear to serve as key indicators of BTV transmission. The data also support the development of vector management and livestock movement control policies tailored to local epidemiological risks.

A major strength of this study is its large, regionally stratified sample size, as well as the inclusion of four economically important livestock species. The use of a validated competitive ELISA with high specificity further enhances the reliability of the seroprevalence estimates. In addition, the study is one of the few recent efforts to characterize BTV exposure in Saudi Arabia, providing valuable insights into a region that has been largely understudied.

However, the study has limitations. The reliance on serological testing prevents the detection of active infections or viral serotypes, and the sensitivity of the assay (82.8%) may underestimate the true prevalence. Furthermore, the absence of concurrent vector surveillance data limits our ability to correlate seroprevalence with *Culicoides* activity.

Future research should incorporate molecular diagnostic tools, such as reverse transcription polymerase chain reaction, to detect circulating BTV strains and assess their genetic diversity. Longitudinal studies are also warranted to monitor seasonal trends and the impact of climate change on vector dynamics. Establishing a national *Culicoides* monitoring system would be instrumental in supporting evidence-based control strategies.

In conclusion, the study highlights the endemic nature of BTV in Saudi Arabia and the disproportionate risk it poses to goats and cattle. These findings provide a foundation for enhancing national animal health policies, mitigating economic losses, and protecting the sustainability of the livestock sector in arid and semi-arid regions.

## DATA AVAILABILITY

All generated data are included in the manuscript.

## AUTHORS’ CONTRIBUTIONS

RAA: Designed and conducted the study, data collection and analysis, interpreted the results, and drafted and revised the manuscript. The author has read and approved the final manuscript.
